# Life-History Plasticity of Cultured *Coreius guichenoti*: Energy Allocation Trade-Offs and Conservation Applications

**DOI:** 10.3390/ani16030456

**Published:** 2026-02-01

**Authors:** Miao Xiang, Haoran Liu, Zihao Meng, Yan Zhao, Chengjie Yin, Xuemei Li, Xingbing Wu, Tingbing Zhu

**Affiliations:** 1 Yangtze River Fisheries Research Institute, Chinese Academy of Fishery Sciences, Wuhan 430223, China; xiangmiaoihb@163.com (M.X.); lhr020620@dingtalk.com (H.L.); mengzh@yfi.ac.cn (Z.M.); zhaoyan@yfi.ac.cn (Y.Z.); xmli@yfi.ac.cn (X.L.); 2 School of Environment and Surveying Engineering, Suzhou University, Suzhou 234000, China; ychj100@163.com; 3 College of Fishery, Huazhong Agricultural University, Wuhan 430070, China

**Keywords:** *Coreius guichenoti*, life-history strategy, plasticity, cultured population

## Abstract

*Coreius guichenoti*, an endemic fish of the upper Yangtze River, has experienced severe population decline due to overfishing and habitat fragmentation. To inform its conservation, this study compared life-history traits between artificially bred and historical wild populations, revealing pronounced plasticity in response to environmental conditions. The cultured population, dominated by age 0–4 individuals but retaining a notable proportion of age 5–6 fish, exhibited faster growth and higher fecundity (mean absolute fecundity 32,724 ± 24,132 eggs; relative fecundity 37.5 ± 18.5 eggs/g) than the wild population. In contrast, the wild group consisted of >90% age 0–4 individuals, showed virtually no fish aged 5–7, reproduced seasonally with high total egg output, and tended toward a periodic life-history strategy, whereas the cultured group tended toward an opportunistic strategy that still retained some periodic traits. These results demonstrate that *C. guichenoti* can adjust its life history on a within-generation scale. Accordingly, we recommend pre-release conditioning with moderate flow and temperature variations to enhance field adaptability. This study provides evidence-based guidance for broodstock selection and preconditioning in restocking programs, aimed at improving post-release survival and reproductive success in the wild.

## Simple Summary

This study examines how captive conditions affect the life-history traits of *Coreius guichenoti*. Fish kept at constant warm temperatures with abundant food tended to grow faster, mature earlier, and produce more and larger eggs than wild fish. The cultured group thus appeared to lean toward an opportunistic strategy focused on rapid reproduction, whereas the wild group tended toward a periodic strategy that balances long life with timed spawning. These cultured traits appear within one generation, showing that the species can flexibly shift energy allocation within a single generation. These findings provide a scientific basis for selecting broodstock and conducting short-term wild acclimatization prior to release, thereby supporting the restoration of this endangered species in the Yangtze River.

## 1. Introduction

Life-history theory categorizes the continuous distribution of fish along a three-dimensional trade-off space defined by generation time, fecundity, and larval survival into three typical strategies: opportunistic, periodic, and equilibrium [1]. The opportunistic strategy is characterized by small body size, early maturation, and a short lifespan; it facilitates rapid population turnover through an extended spawning season, production of numerous small eggs, and minimal parental investment [2,3]. This strategy predominates in habitats with pronounced hydrological variability, frequent droughts, or substantial anthropogenic disturbance, where populations exhibit marked fluctuations yet demonstrate rapid recovery following severe perturbations [4]. In contrast, the periodic strategy features slow growth, large body size, extended longevity, and delayed maturation. Individuals release large quantities of small eggs during a restricted spawning window, with high larval mortality [5]. This approach depends on predictable seasonal flooding for synchronized mass spawning, employs long lifespans and high fecundity to buffer periods of low recruitment, tolerates moderate drought conditions, but is vulnerable to environments with high interannual variability [6]. The equilibrium strategy is distinguished by low absolute fecundity, large egg size, and a prolonged reproductive season. Species adopting this strategy often exhibit brood care behaviors such as nest building, egg guarding, or mouthbrooding, and are typically associated with stable environments such as deep lakes or regulated river reaches with consistent flow regimes [7]. Populations following this strategy show low interannual variability and remain relatively stable even under recurrent flooding or reduced carrying capacity [8]. Most fishes, however, exhibit trait combinations intermediate to these three archetypal strategies, forming a continuum of phenotypes aligned with environmental gradients [9]. Such intermediate phenotypes can adjust resource allocation within a single generation in response to shifts in temperature, resource availability, or predation pressure [10,11], a response known as life-history plasticity [12,13]. Quantifying the magnitude and trajectory of life-history plasticity is essential for predicting how fish populations will respond to ongoing and future environmental changes.

Growth and reproduction represent two fundamental axes of fish life history, with associated traits determining population renewal rates and resource allocation strategies [14]. Growth is modulated by water temperature, food availability, and predation pressure through their effects on metabolic rate and energy assimilation. Under warm, stable conditions with abundant resources, fish can rapidly approach their maximum potential size, whereas frequent environmental disturbances or elevated predation risk reduce growth coefficients and prolong generation time [15]. Reproduction exhibits comparable environmental sensitivity, as traits such as egg size, absolute fecundity, and age at first maturation vary in response to changes in energy availability within the environment [16]. In aquaculture systems, constant temperatures, high food abundance, and the absence of predation establish a stable habitat that promotes the reallocation of energy from defensive and escape behaviors toward growth and reproductive investment. Under these conditions, parents allocate more energy to somatic maintenance while simultaneously increasing reproductive effort, often producing larger eggs, as they anticipate higher offspring survival [17]. In contrast, wild populations subject to heavy fishing or altered flow regimes may raise reproductive output and reduce egg size, trading offspring quality for quantity as a means of compensating for unpredictable survival risks [18,19]. The phenotypic divergence between cultured and wild groups thus offers a controlled framework, free from external gene flow, to quantify the extent to which environmental conditions shape energy allocation patterns. Such insights can inform the establishment of temporal and intensity thresholds in conservation planning and help mitigate the loss of field fitness in stocked fish due to maladaptation.

*Coreius guichenoti* is an endemic species of the upper Yangtze River. Historically, it constituted over half of the local catch, serving as a cornerstone of the regional fishery and possessing significant ecological and economic value [20]. Over the past two decades, however, its population has experienced a severe decline. Overfishing has led to reductions in body size and a shift toward younger age classes, while large dams have obstructed migratory pathways [21,22]. The conversion of flowing water to reservoir conditions has eliminated the drifting incubation environment, drastically reducing spawning success [23]. Furthermore, cascades of hydropower stations have inundated spawning grounds, compressing and fragmenting the remaining habitat [24]. Several studies indicate that under the combined pressures of overfishing and habitat degradation, the wild population has been reduced to an ecologically precarious level [24,25]. Consequently, the species is now classified as critically endangered on the China Vertebrate Red List, and its wild population is listed under Class II of the National Key Protected Wildlife Catalogue. Although the ten-year fishing moratorium has alleviated direct harvest pressure, it is insufficient to restore natural recruitment in the short term [26,27]. As a result, captive breeding and stock enhancement have become essential for sustaining genetic connectivity. Successful full artificial propagation has enabled the establishment of a stable captive population [26]. Comparing growth and reproductive traits between the current cultured stock and historical wild data can elucidate how anthropogenic disturbances have altered the species’ life-history strategy. Such analyses will provide quantitative benchmarks for guiding future stocking practices, optimizing age structure, and setting habitat restoration targets.

This study quantifies life-history plasticity in cultured *C. guichenoti* under controlled conditions and compares it with historical wild populations. By using a stable, artificially propagated stock, we track within-generation phenotypic shifts in a single captive cohort, thereby reducing the influence of long-term evolutionary signals. The results guide the integration of plasticity into stocking and broodstock management to boost the ecological adaptability of released fish.

## 2. Materials and Methods

### 2.1. Fish Maintenance

The Yangtze River Fisheries Research Institute of the Chinese Academy of Fishery Sciences has achieved significant advances in the artificial propagation of *C. guichenoti*. In 2013, wild broodstock were successfully induced to spawn for the first time, and the resulting larvae were reared to maturity in a recirculating aquaculture system (RAS). The following year, broodstock raised in the same system were again induced to spawn, yielding eggs and larvae that were subsequently cultured to the juvenile stage. By 2016, batch maturation of broodstock and small-scale seed production had been accomplished within the RAS framework. To date, the institute has maintained successful artificial propagation for ten consecutive years, establishing a stable, controllable, large-scale breeding system capable of producing over 100,000 viable juveniles annually.

**Figure 1:**
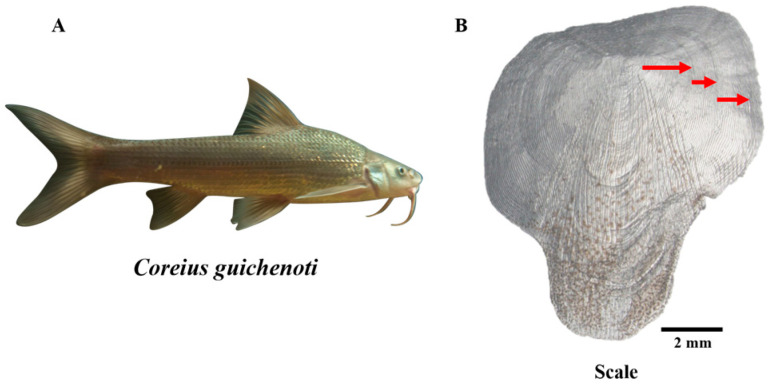
*Coreius guichenoti* (**A**) under rearing conditions and scales used for age determination (**B**). Note: the red arrows indicate the annuli, showing that this individual is 3 years old.

Since 2016, the team has used RAS to rear first-generation captive broodstock, with more than 5000 *C. guichenoti* breeders of varying ages currently maintained (Figure 1A). The broodstock are housed in a closed RAS situated within a climate-controlled greenhouse. The system includes two cylindrical tanks, each with a diameter of 1.8 m, a height of 1.0 m, and a water depth of 0.78 m, with water exchanged once per hour. Throughout the rearing period, water temperature is maintained below 20 °C. Fish are fed a floating pellet diet twice daily (at 09:00 and 15:00), containing ≥42% crude protein, ≥5% crude fat, and ≤12.5% moisture. Daily ration ranges seasonally from 0.5% to 2% of body weight, provided to satiation [28]. Each breeding season, gonadal and other tissue samples are systematically collected and preserved for reproductive analyses. Any mortalities occurring during routine husbandry are promptly removed, preserved using standardized protocols, and utilized for growth measurements and further study.

### 2.2. Age Structure and Growth Pattern

All individuals were measured for standard length (SL, to the nearest 0.1 cm) and body weight (BW, to the nearest 0.1 g). For age validation in fish, our aquaculture facility employs a standardized protocol that begins with the artificial propagation and rearing of wild-sourced populations. The spawning and hatching dates of each cohort are systematically recorded and managed on a year-class basis, enabling precise age determination for all individuals within a given cohort. In addition to cohort records, scale analysis was used as a complementary method for age verification. Scales were collected from the region near the pectoral fin insertion, promptly rinsed, and soaked in a 0.5% sodium hydroxide solution for several hours to remove adhering tissue. After cleaning, scales were thoroughly rinsed with freshwater, air-dried, and mounted on microscope slides. Age readings were conducted independently by the first author, with three repeated assessments performed at minimum intervals of two weeks to ensure consistency and reduce observer bias (Figure 1B). Age was assigned based on the number of annual rings (annuli) observed on each scale: fish with no annulus were classified as age 0, one annulus as age 1, two annuli as age 2, and so forth [19].

To estimate growth parameters, the age-length data were fitted to the following three candidate growth models:

The Von Bertalanffy growth function (VBGF):

$${\mathrm{SL}}_{t}\;={\;\mathrm{SL}}_{\infty }\left(1\;-\;e^{-K\left(t\;-\;t_{0}\right)}\right)$$

The Logistic growth function (Logistic GF):

$${\mathrm{SL}}_{t}\;=\;{\mathrm{SL}}_{\infty }/\left(1\;+\;e^{-K\left(t\;-\;t_{0}\right)}\right)$$

The Gompertz growth function (Gompertz GF):

$${\mathrm{SL}}_{t}\;=\;{\mathrm{SL}}_{\infty }e^{-e^{-K(t\;-\;t_{0})}}$$

In each model, ${SL}_{t}$ is the length at age *t*, ${SL}_{\infty }$ is the asymptotic length, *K* is the mean curvature of the growth curve, and $t_{0}$ is the age of the theoretical growth starting point. Each model was fitted using the Gauss–Newton algorithm to minimize the residual sum of squares [29]. Model selection was performed based on Akaike’s Information Criterion (AIC) and the Bayesian Information Criterion (BIC), with the model exhibiting the lowest values of both criteria considered the most appropriate [30].

### 2.3. Fecundity

The classification of gonadal stages (IV and V) was based on macroscopic morphological characteristics, following the criteria established for wild populations by Yang et al., 2018 [31]. Stage IV (Mature/Pre-spawning): ovaries appear bluish-gray with numerous visible blood vessels; oocytes exhibit substantial yolk deposition. Stage V (Ripe/Spawning): Blood vessels on the ovaries are reduced; oocytes become slightly translucent, round, and easily dispersed within the ovarian cavity, with eggs flowing freely under gentle abdominal pressure. Individuals at stages IV and V were dissected for fecundity analysis. The gonads (gonad weight, W_G_) were weighed. After confirming comparable size between the left and right ovaries, approximately 0.3 g of oocytes were collected as sub-samples from the anterior, middle, and posterior regions of the right ovary. The total number of oocytes (*n*) in each sub-sample was counted under a dissecting microscope (Olympus SZX16, Olympus Corporation, Tokyo, Japan) under visible light at 2× magnification. The oocytes were then homogenized, weighed (Wo), and fixed in 5% formaldehyde. Absolute fecundity (AF) was calculated using the following formula:

AF = *n* × W_G_/W_O_

relative fecundity (RF) as:

RF = AF/BW

### 2.4. Data Analysis

Owing to the species’ protected status and current fishing ban policies, obtaining wild populations of this fish is presently challenging. To evaluate long-term shifts in life-history traits, we compared the cultured stock with historical wild populations of the upper Yangtze River, i.e., the population in 2004–2007 [22] and the population in 2013–2014 [31]. We performed principal component analysis (PCA) to examine temporal shifts in life-history traits of fish populations in the upper Yangtze River. The analysis incorporated key parameters including egg size, absolute fecundity (AF), relative fecundity (RF), age at first maturity, and asymptotic standard length (${\mathrm{SL}}_{\infty }$) in the catch of each population. Based on the classic life-history framework and PCA methodology established by Winemiller and Rose [1], these variables were reduced to two principal components for further interpretation. All life-history variables were standardized using Z-score normalization to eliminate scale differences and meet the variance comparability requirement of PCA.

Age at first maturity was estimated as the age at which 50% of individuals were mature (stages IV–V) by fitting a logistic regression to the proportion mature across 0.5-year age classes. For the cultured stock, quarterly random samples were taken during the expected maturation period; the youngest age at which any fish reached stage IV or V was recorded as the earliest maturation time.

Linear regression was used to examine relationships between absolute fecundity and both body length and body weight. All datasets were initially assessed for normality; when transformation did not achieve normality, non-parametric tests were applied. Results are expressed as mean ± standard deviation (SD). Statistical analyses and graphical presentations were conducted using SPSS 19.0 and Origin 2021.

## 3. Results

### 3.1. Standard Length and Body Weight of Coreius guichenoti

**Figure 2:**
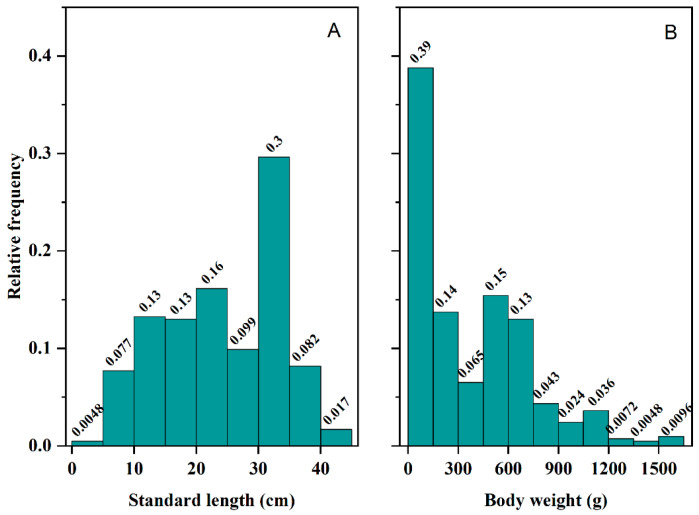
Frequency distributions of standard length (**A**) and body weight (**B**) of *Coreius guichenoti* under rearing conditions.

A total of 415 cultured *C. guichenoti* were examined. Standard length ranged from 4.5 to 43.5 cm, with a mean of 24.0 ± 9.6 cm; body weight ranged from 1.5 to 1545.0 g, with a mean of 372.8 ± 345.7 g. More than half of the individuals measured between 10 and 30 cm in length, and the length class of 30–35 cm showed the highest relative frequency. Fish shorter than 5 cm or longer than 40 cm were rarely observed. In terms of weight, over 50% of the sample fell within the 0–750 g range, with the 0–150 g class being the most prevalent, whereas individuals heavier than 1200 g were uncommon (Figure 2). Wild reference values (mean ± SD: 14.8 ± 6.25 cm SL, 147.8 ± 62.5 g BW) are cited for cross-study comparison; sampling seasons may differ [22].

**Figure 3:**
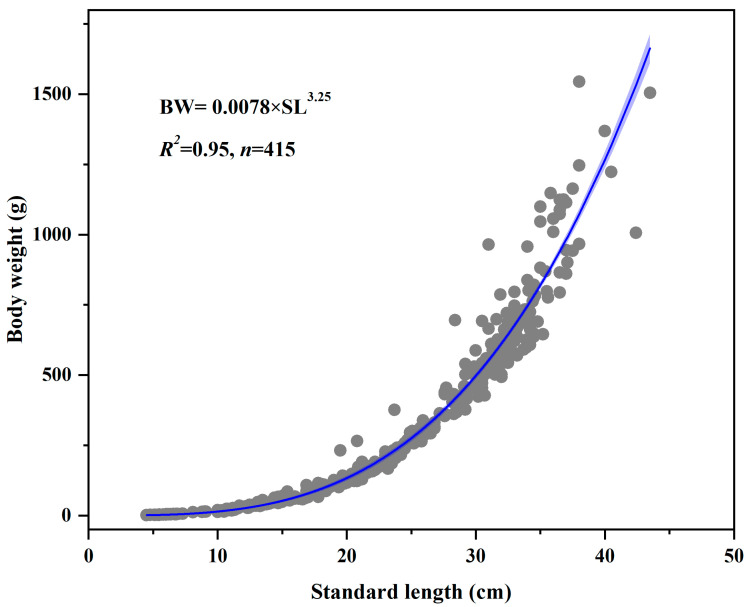
Length–weight relationship of *Coreius guichenoti* under rearing conditions.

The length–weight relationship across the 415 individuals followed BW = 0.0078 SL^3.25^ (R^2^ = 0.95, *n* = 415) (Figure 3). Wild reference (BW = 1 × 10^−5^ SL^3.0311^) is cited for cross-study comparison; sampling seasons may differ [22].

### 3.2. Age and Growth of Coreius guichenoti

**Figure 4:**
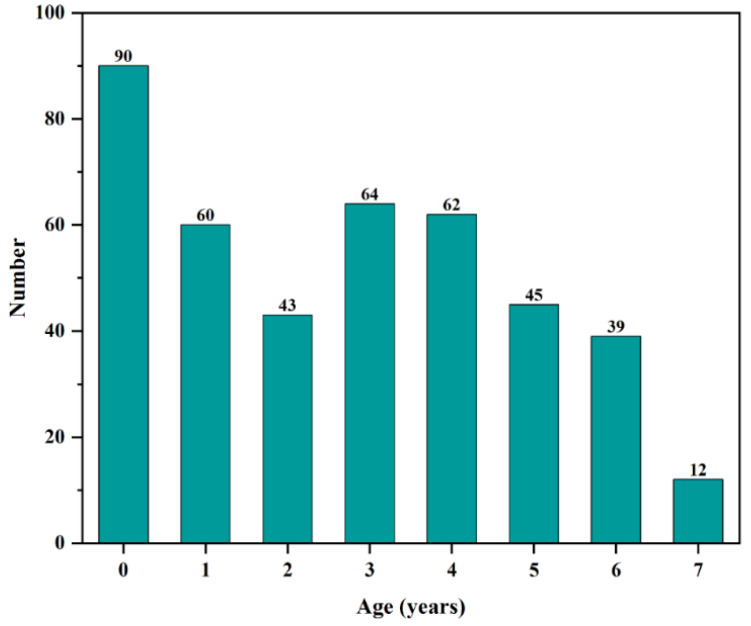
Age structure of *Coreius guichenoti* under rearing conditions.

The age structure of the cultured *C. guichenoti* population ranged from 0 to 7 years, with 7 years representing the maximum recorded age. The distribution was skewed toward younger age classes, in which low-age individuals predominated, while fish approaching the maximum age were scarce (Figure 4).

Among the three candidate growth models, the von Bertalanffy equation exhibited the lowest AIC and BIC values along with the highest R^2^ and was, therefore, selected as the best-fitting model for describing growth in this population. The Gompertz and logistic models showed comparable AIC and BIC values with moderately lower R^2^ and remain suitable as alternative or supplementary models that still provide reasonable descriptions of the observed growth patterns (Table 1).

**Figure 5:**
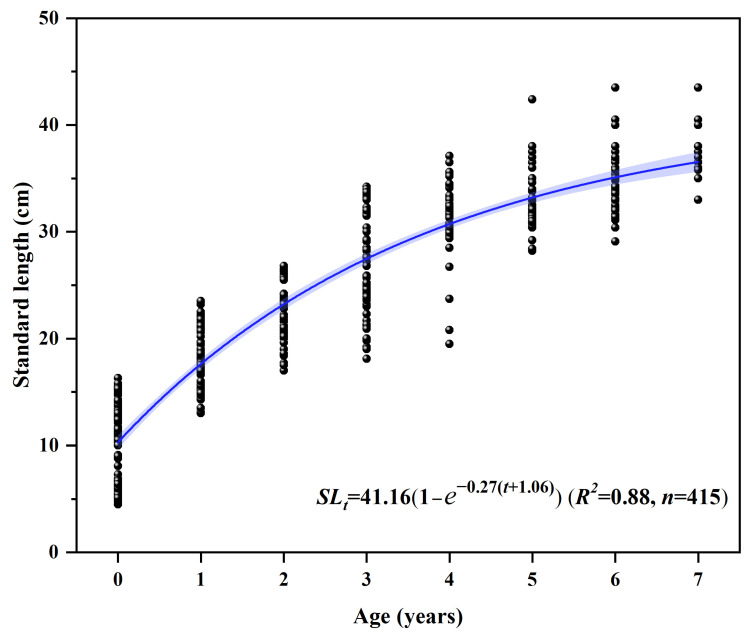
Von Bertalanffy curve of *Coreius guichenoti* under rearing conditions.

Therefore, the Von Bertalanffy growth equation was adopted to describe the relationship between standard length (SL) and age (t): ${\mathrm{SL}}_{t}\;=\;41.16\left(1\;-{\;e}^{-0.27\left(t\;+\;1.06\right)}\right)$ (R^2^ = 0.88, *n* = 415) (Figure 5). Wild reference parameters (${\mathrm{SL}}_{t}\;=\;72.04\left(1\;-e^{-0.1096\left(t\;+\;0.7788\right)}\right)$) are cited for cross-study comparison; sampling seasons may differ [22].

### 3.3. Fecundity of Coreius guichenoti

During the study, 64 mature *C. guichenoti* were collected for fecundity analysis. Their standard length ranged from 27.6 to 43.5 cm (mean ± SD: 33.7 ± 3.4 cm), and body weight ranged from 422.8 to 1545.0 g (mean ± SD: 783.4 ± 272.0 g). Absolute fecundity varied from 3423 to 105,904 eggs (mean ± SD: 32,724 ± 24,132 eggs), and relative fecundity ranged from 7.94 to 74.46 eggs/g (mean ± SD: 37.48 ± 18.50 eggs/g).

**Figure 6:**
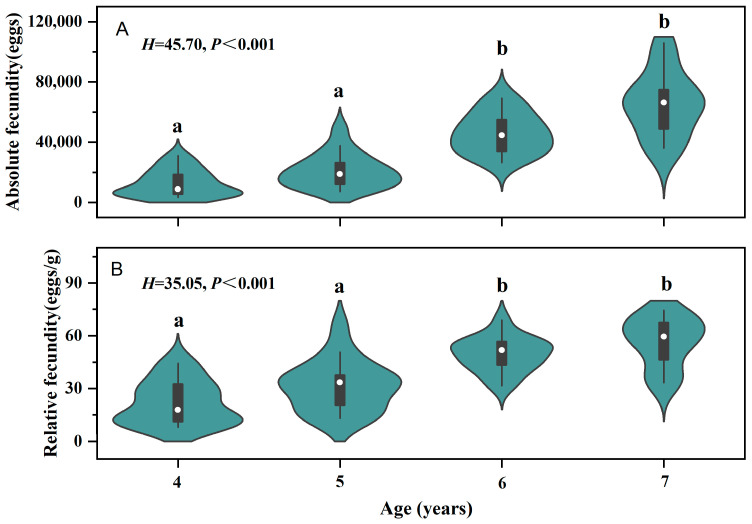
(**A**) Comparison of absolute fecundity (AF) among different age groups of *Coreius guichenoti*; (**B**) Comparison of relative fecundity (RF) among different age groups of *Coreius guichenoti*. Note: different letters (a, b) indicate statistically significant differences (*p* < 0.05).

Age-specific fecundity analysis showed that four-year-old individuals had a mean absolute fecundity of 12,776 ± 8723 eggs and a mean relative fecundity of 21.86 ± 12.26 eggs/g (*n* = 19); corresponding values for five-year-olds were 20,806 ± 11,164 eggs and 31.34 ± 14.32 eggs/g (*n* = 18), for six-year-olds 46,020 ± 13,247 eggs and 49.40 ± 9.81 eggs/g (*n* = 15), and for seven-year-olds 65,566 ± 21,388 eggs and 56.55 ± 14.17 eggs/g (*n* = 12). Kruskal–Wallis tests indicated significant differences among age groups in both absolute fecundity (*H* = 45.70, *p* < 0.001) and relative fecundity (*H* = 35.05, *p* < 0.001). Dunn’s post hoc test with Bonferroni correction revealed that older spawners (ages 6–7) exhibited significantly higher fecundity values than younger spawners (ages 4–5) (Figure 6).

**Figure 7:**
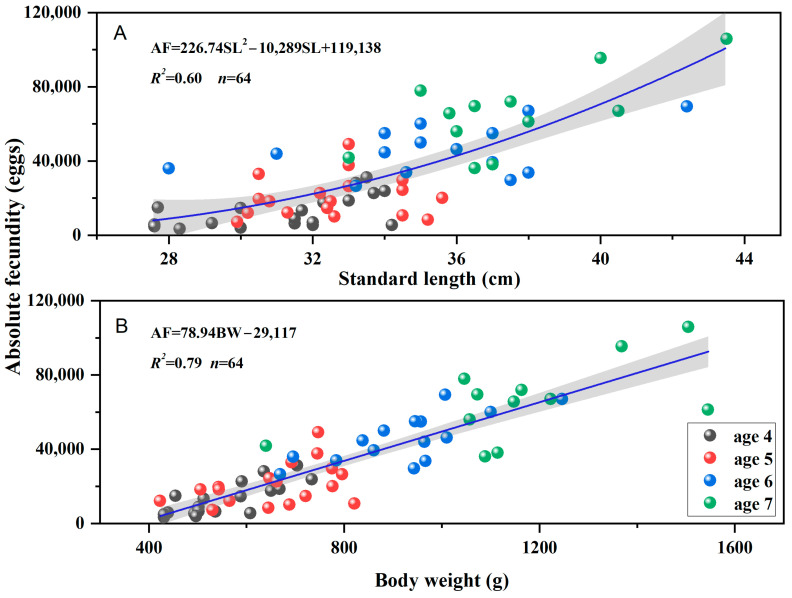
Relationships of absolute fecundity to standard length (**A**) and body weight (**B**) in different age classes for *Coreius guichenoti* (black, age 4; red, age 5; blue, age 6; green, age 7).

The results indicate that absolute fecundity increases markedly with age, standard length, and body weight (Figure 7). The relationship with standard length was best described by the quadratic model: AF = 226.74SL^2^−10,289 × SL + 119,138(*R^2^* = 0.60, *n* = 64), while the relationship with body weight followed a linear model: AF = 78.94BW − 29,117(*R^2^* = 0.79, *n* = 64).

### 3.4. Changes in Life-History Traits of Coreius guichenoti

**Figure 8:**
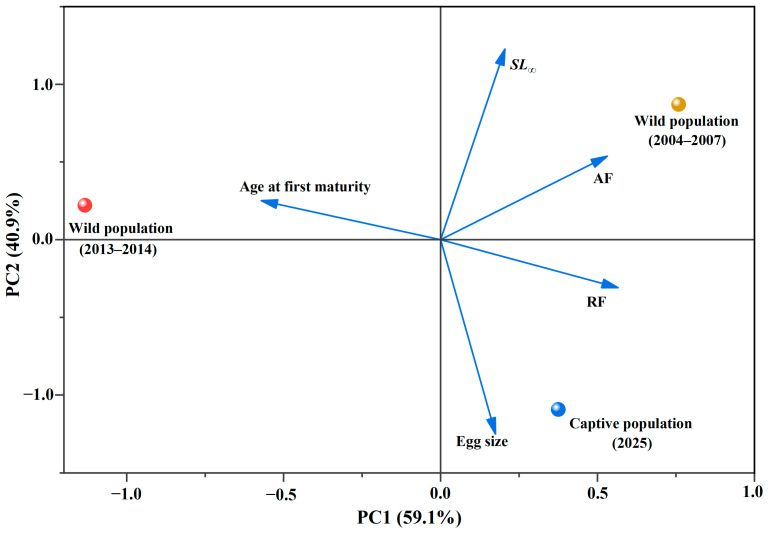
Temporal changes in the life histories of *Coreius guichenoti* in the upper Yangtze basin as revealed through exploratory PCA. Note: this PCA is based on group-level means and is used for visualizing trait differences.

A PCA was built that explained 100% of the variation in the five life-history traits examined (Figure 8). PC1 and PC2 explained 59.1% and 40.9% of the variance, respectively. PC1 was positively driven by asymptotic length (*SL_∞_*) and absolute fecundity (AF) and negatively driven by age at first maturity. In contrast, PC2 was negatively driven by egg size and relative fecundity (RF). Thus, the cultured samples were characterized by larger egg size, higher relative fecundity, smaller asymptotic length, and lower absolute fecundity compared to the two historical populations (Figure 8, Table A1).

## 4. Discussion

### 4.1. Comparative Analysis of Growth and Energy Allocation in Cultured vs. Wild Coreius guichenoti

Compared with the historic wild population, the cultured population exhibited faster growth and a younger age structure. Body length distribution was right-shifted, with cultured fish averaging 24.0 ± 9.6 cm (approximately 30% falling within the 30–35 cm size class), whereas wild fish averaged only 14.8 ± 6.25 cm (mostly between 10 and 14 cm) [22,31]. The disparity in body weight was even more pronounced: cultured individuals averaged 372.8 ± 345.7 g, about 2.5 times the wild mean of 147.8 ± 62.5 g. While 40% of the cultured fish weighed less than 150 g, over half of the wild fish weighed below 50 g. Both groups displayed strong length–weight relationships (R^2^ ≥ 0.95); however, the allometric exponent was higher in the cultured group (3.25) than in the wild (3.03), indicating that cultured fish attained greater mass at comparable lengths [22,31]. The age composition also differed markedly. In the cultured sample, age 0 to age 4 individuals predominated, age 5 and age 6 fish remained relatively common, and the proportion dropped sharply at age 7. In contrast, more than 90% of the wild population consisted of age-1 to age-4 fish, with individuals aged 5–7 virtually absent [22,31].

The accelerated growth observed in cultured *C. guichenoti* was primarily driven by three factors: diet, temperature regime, and reduced activity-related energy expenditure. Continuous provisioning of high-protein feed throughout the year resulted in daily energy intake substantially exceeding estimates for wild populations, which rely on plankton and benthos [32,33]. This stable energy supply, combined with lower swimming costs in the culture environment, increased the daily energy surplus and promoted the allocation of protein toward somatic growth and visceral fat accumulation [34]. Consequently, body weight increased more rapidly than length, elevating the allometric exponent from 3.03 in the wild to 3.25 and improving overall physiological condition [35,36]. Maintaining water temperature consistently below 20 °C simulated the effective accumulated temperature typically observed during the spring growth peak in the wild, thereby eliminating seasonal growth retardation and associated energy losses [37]. This regime contributed to an increase in the von Bertalanffy growth coefficient (K) to 0.27, markedly higher than the reported wild value of 0.11, indicating that cultured individuals approach their asymptotic length at an earlier age. Furthermore, the absence of predation and low water velocity in culture systems reduced routine metabolic costs, channeling additional energy into biomass accumulation [32,36]. Nevertheless, both cultured and wild populations exhibited few individuals at the extremes of size, suggesting that genetic and physiological constraints define a bounded growth window for the species [38,39]. In natural habitats, seasonal food limitation and fishing pressure likely prevent most wild fish from surviving beyond age 5. Under culture conditions, the lack of predation and abundant energy allow a higher proportion of individuals to reach ages 5–6; however, the sharp decline in abundance at age 7 implies that intrinsic senescence or physiological exhaustion occurs as the species approaches its maximum lifespan and that additional energy input cannot extend ultimate longevity [39,40]. Thus, culture conditions appear to unlock midlife growth potential without overriding the species’ inherent life-span ceiling. Notably, despite superior growth performance, cultured fish accumulated higher body fat, which may compromise swimming endurance and reduce post-release survival when encountering natural challenges such as high flow velocities and predators [41]. Moreover, constant temperature regimes preclude thermal acclimatization, potentially diminishing the capacity to cope with natural temperature variability [34]. Therefore, rapid growth under culture is not an unqualified advantage. Future conservation-oriented propagation protocols should consider establishing upper limits for feed ration and temperature, while incorporating phased temperature and flow-velocity conditioning. Such measures would help maintain field-relevant fitness during accelerated growth, thereby preventing culture-acquired traits from becoming liabilities after stocking.

### 4.2. Reproductive Energy Allocation Across Environments and the Age Threshold

*C. guichenoti* reaches sexual maturity at approximately age 4 in both cultured and wild populations. Cultured individuals matured at a standard length of 27.6–43.5 cm (mean 33.7 cm), close to the lower limit observed in the wild, yet exhibited greater energy reserves at comparable body lengths. After surpassing the length threshold for maturation, surplus energy is directly allocated to ovarian development, leading to a substantial increase in overall fecundity [42]. Both absolute and relative fecundity rose significantly with age (Kruskal–Wallis *H* = 45.70 and 35.05, respectively; *p* < 0.001), with individuals aged 6–7 showing markedly higher values than those aged 4–5, indicating that energy reserves are the primary determinant of elevated egg production [43]. Based on the triangular life-history model, principal component analysis revealed a separation trend between the cultured population and historical wild samples within the multivariate trait space. The cultured population shifts toward a strategy characterized by high fecundity, large egg size, and reduced asymptotic length, whereas the wild groups historically exhibited a more periodic strategy defined by a short spawning season and high total egg output. This transition trend suggests that the reproductive advantage observed under culture conditions may arise not only from larger body size but also from a possible reallocation of life-history trade-offs when energy supply is both predictable and abundant. Compared with wild fish, cultured fish slightly reduce the size at first maturity and appear to reallocate energy that would otherwise support somatic growth into gonadal investment. Increased relative fecundity compensates for the reduced asymptotic length, and a modest increase in egg diameter indicates that nutrient investment per oocyte is maintained even as egg number rises—thereby avoiding the typical trade-off between egg quantity and quality. Collectively, these adjustments lean toward the opportunistic end of the continuum while retaining some periodic traits.

Specifically, the cultured group exhibited an absolute fecundity of 32,724 ± 24,132 eggs and a relative fecundity of 37.5 ± 18.5 eggs/g, both notably higher than published values for wild populations. At each age class from 4 to 7 years, relative fecundity in the cultured fish (21.86, 31.34, 49.40, and 56.55 eggs/g) exceeded that reported for wild counterparts (approximately 17, 18, 21, and 45 eggs/g, respectively), with the difference widening progressively with age. A continuous provision of high-protein feed combined with reduced swimming costs generated a sustained energy surplus, thereby allocating a greater resource budget to ovarian development in each reproductive cycle [44]. Moreover, consistently warm water temperatures and extended daily photoperiod prolonged the rapid-growth phase of gonad maturation and minimized winter oocyte atresia [32,34], further increasing the number of viable eggs. In contrast, wild fish must contend with seasonal fluctuations in food availability and elevated metabolic costs during winter, necessitating a conservative balance between reproductive investment and somatic maintenance that constrains fecundity below its physiological potential [45]. Additionally, culture conditions-including controlled stocking density, absence of predators, and lower circulating cortisol levels [36,46] may alleviate environmental and physiological constraints on gonad development. The high nutritional quality of formulated feed also enhanced the synthesis of yolk precursors, leading to improvements in both egg quantity and quality.

With advancing age, cultured females are not subject to fishing mortality and thus do not require extensive somatic investment in cruising musculature. This allows them to reallocate a greater proportion of protein reserves from somatic tissues to gonadal development, establishing a positive feedback loop in which both body size and reproductive output increase concurrently. In contrast, older wild females must contend with elevated maintenance costs in fast-flowing habitats and are disproportionately targeted by size-selective fishing. These constraints limit the continuous allocation of surplus energy to the ovaries, causing population-level fecundity to plateau. Consequently, under artificial conditions, reproductive output may be enhanced concurrently through relatively high energy intake, low locomotor expenditure, and reduced physiological stress. Nevertheless, even by age 7, fecundity values in the cultured population do not surpass the maximum recorded in wild individuals. Therefore, it is recommended that broodstock programs prioritize egg collection from females aged 5–6 years, when fecundity increases most rapidly and egg size peaks, rather than relying on older individuals that may carry excessive somatic fat with potential fitness trade-offs.

### 4.3. Life-History Plasticity and Its Value for Conservation

This study demonstrates that cultured and wild *C. guichenoti* occupy distinct regions within the life-history trait space, indicating that the species can reallocate energy allocation patterns within a single generation. Although genetic change was not evaluated, cultured fish consistently express a phenotype characterized by high fecundity, larger egg size, and smaller asymptotic length, all of which align with the features of life-history plasticity [47,48]. The findings further quantify how the species maximizes population growth via plasticity when environmental stressors shift from predation, food limitation, and winter cold to conditions of consistent high nutrition, stable temperature, and absence of predators. This phenotypic transition is fully replicable under controlled culture conditions, providing a tractable experimental system for conservation-oriented research. Future work should focus on identifying the optimal age structure and nutritional thresholds for broodstock destined for release while also incorporating male maturation and reproductive data to refine selection protocols, thereby avoiding unnecessary reductions in effective population size that may result from non-selective spawning practices.

As plasticity is bounded by physiological limits, fecundity no longer increases linearly with energy input beyond age 7, suggesting that ovarian capacity or endocrine regulation has reached an intrinsic ceiling. Further delaying reproduction only elevates individual mortality risk without yielding significant reproductive returns [49]. Therefore, conservation programs should prioritize collecting high-quality eggs from females aged 5–6 years to ensure sufficient offspring numbers. To compensate for the reduced swimming stamina and thermal tolerance resulting from accelerated growth, the F1 generation can be periodically transferred to semi-natural channels where controlled, staged fluctuations in flow and temperature simulate natural seasonal variability [50,51]. This approach maintains high reproductive output while reinstating behavioral and physiological flexibility, thereby enhancing post-release survival and accelerating population recovery. Owing to the protected status of *C. guichenoti* and the fish ban, cross-study comparisons may be influenced by differences in sampling season and site. Future studies should integrate genomic and physiological markers to elucidate the pathways governing energy allocation, establish a post-release recapture monitoring system to evaluate whether the growth and fecundity parameters identified here remain consistent across different years and river reaches, and continue to track annual gonadosomatic index (GSI) dynamics in males to clarify seasonal reproductive investment and its influence on population renewal.

## 5. Conclusions

Cultured *C. guichenoti* exhibits a life-history profile of high fecundity, larger egg size, and reduced asymptotic length. The population is dominated by young individuals that allocate surplus energy primarily to somatic growth and gonadal development, resulting in a plumper body condition at comparable lengths and the production of larger eggs. In contrast, the wild population aligns more closely with a periodic strategy, characterized by synchronized spawning within a constrained season and a comparatively high total egg output. The cultured group, however, displays a tendency toward an opportunistic life-history trajectory while retaining certain periodic reproductive traits. Although cross-study comparisons may be influenced by differences in sampling season and site, we minimized this limitation by applying the same measurement criteria and modeling procedures used in the historical surveys. By actively managing this directional plasticity, conservation programs can target ages 5–6 as the optimal window for both egg collection and stock release. Integrating staged temperature and flow-variation training into rearing protocols will help maintain high reproductive potential while gradually rebuilding tolerance to natural thermal fluctuations, hydrodynamic challenges, and predation pressure. Such an approach is expected to enhance the post-release persistence of cultured stocks and improve their capacity for self-sustenance in wild environments.

## Figures and Tables

**Table 1:** Estimated growth function parameters for SL-at-age and AIC and BIC for *Coreius guichenoti* under rearing conditions.

Growth Function	${SL}_{\infty }$ (cm)	*K* (Year^−1^)	*t*_0_ (Years)	*AIC*	*BIC*	*R* ^2^
VBGF	41.16	0.27	−1.06	1002.31	1018.43	0.880
Logistic GF	37.56	0.68	1.22	1014.66	1030.77	0.877
Gompertz GF	36.03	0.47	0.50	1006.27	1022.38	0.879

## Data Availability

The data presented in this study are available on request from the corresponding author.

## Appendix A

**Table A1:** Data used in the PCA include historical populations and cultured populations.

Year	Egg Size (mm)	AF (Eggs)	RF (Eggs/g)	Age at First Maturity (Year)	Asymptotic Length (cm)	Reference
2025	1.94 ± 0.08	32,724 ± 24,132	37.48 ± 18.50	4.00	41.16	this study
2004–2007	1.59 ± 0.31	46,386 ± 51,589	36.6 ± 27.65	4.00	72.04	Cheng, 2008 [22]
2013–2024	1.60	22,817.00	20.00	4.32	52.08	Yang et al., 2018 [31]
